# Show me the money: how do we justify spending health care dollars on digital health?

**DOI:** 10.5694/mja2.51799

**Published:** 2022-12-11

**Authors:** Leanna Woods, Rebekah Eden, Oliver J Canfell, Kim‐Huong Nguyen, Tracy Comans, Clair Sullivan

**Affiliations:** ^1^ Centre for Health Services Research University of Queensland Brisbane QLD; ^2^ Queensland Digital Health Centre University of Queensland Brisbane QLD; ^3^ Digital Health Cooperative Research Centre Sydney NSW; ^4^ Queensland University of Technology Brisbane QLD; ^5^ University of Queensland Brisbane QLD; ^6^ Global Brain Health Institute Trinity College Dublin and University California San Francisco Dublin Ireland; ^7^ Royal Brisbane and Women's Hospital Metro North Hospital and Health Service Brisbane QLD

**Keywords:** eHealth, Information management, Health financing, Health services research

Focusing solely on financial measures is unlikely to deliver a comprehensive view of the value of digital health

Digital health, which refers to the use of digital technology to provide and support health care services, promises to strengthen health systems worldwide and has been accelerated by the coronavirus disease 2019 (COVID‐19) pandemic.^1^ Amid the rapid digital transformation of health care,^2^, ^3^ the value of sizeable digital health investments remains unclear.^3^, ^4^ The investments required for digital health are often substantial and may come at a cost to existing health care delivery models. Decision makers can be paralysed by a situation where investment in digital health is unavoidable but the conventionally measured short term outcomes often do not provide a convincing fiscal return on investment.^2^, ^3^

However, health systems are now shifting from perceiving digital health as a cost that needs to be recouped to a quality improvement tool that can positively transform health care. This is because large‐scale digital health implementations, such as electronic medical records (EMRs), provide significant quality and safety benefits, including:^5^

- reduced unwarranted variation in care;
- reduced preventable harm;
- improved patient centredness; and
- enhanced opportunities for monitoring, risk management, and quality improvement.

These quality and safety benefits, despite being significant and meaningful to clinicians and consumers, are difficult to cost, rarely deliver a rapid financial benefit to the funder, and are traditionally not measured in financial evaluations.

To maximise the potential of digital health, a more sophisticated approach than simple monetary return on investment needs to be developed and adopted. The quadruple aim of health care — better population health outcomes, improved care experience for patients, improved work life of health professionals, and reduced health care costs^6^ — is a straightforward and increasingly adopted approach for evaluating quality improvement interventions in health care.

In this perspective article, we show the potential of the quadruple aim to transform how digital health is measured and so how it is valued. Using the quadruple aim as a streamlined, scalable framework to quantify the real‐world impact of digital transformations allows a new level of granularity that enables continuous and clinically meaningful improvement. We present a case study of applying the quadruple aim framework to understand the value of a hospital‐based EMR, delivering 65% of all public health care across 16 digital hospitals in Queensland, Australia.

## Measuring health system performance: from business cases to the quadruple aim

Criteria for evaluating the performance of health care have evolved since *The world health report 2000* initially identified three fundamental criteria to measure the performance of health systems: good population health, the ability to respond to consumers’ expectations, and fairness of financial contribution.^7^ In 2001, the Institute of Medicine published consensus‐based measures to evaluate functioning health care systems that are safe, effective, patient‐centred, timely, efficient and equitable.^8^ Then in 2008, the Institute for Healthcare Improvement^9^ proposed pursuit of the triple aim: improving patient experience, improving health, and reducing the cost of care. This was later challenged in an article published in 2014 which argued that positive engagement of the health workforce was paramount to achieving the triple aim.^6^ The fourth dimension — improving the experience of the health care workforce — was added. The quadruple aim has been adopted in health care workforce,^10^, ^11^ innovation implementation,^12^ and COVID‐19 pandemic^13^ contexts to drive a balanced scorecard for health care projects, forcing health care funders to look beyond traditional fiscal return. The discourse has evolved in 2022 to include health equity to form the quintuple aim.^14^

## Existing digital health evaluation models

Recent systematic reviews have highlighted multiple strategies to assess the impact of digital health to inform future investments^15^, ^16^ and have reported challenges due to the heterogenous, emerging and uncertain nature of digital health impacts.^15^, ^17^, ^18^ A range of models are currently in use to evaluate digital health (Box 1).

Box 1 **Sample of digital health evaluation methods in current use**

**Table:** 

**Existing approach**	**Description**	**Strengths**	**Weaknesses**
Economic evaluations (eg, return on investment)^4^, ^19^	Benefit minus the cost expressed as a proportion of the cost	Value the financial return of a business investment	Focus on financials only; limited to comparison to existing services^4^
Canada Health Infoway's Benefits Evaluation Framework^18^	Proposes that technical attributes of the system (ie, system, information and service quality) influence how the system is used and staff satisfaction with the system, which in turn influences the benefits attained	Comprehensive measures evaluating benefits of digital health linked to quality of outcomes, patients’ access to services, and productivity improvements	Workforce implications are overlooked
Electronic Medical Record Adoption Model, Healthcare Information and Management Systems Society^20^	Evaluates the maturity of an organisation based on the extensiveness of their digital health investments	Provides a tool for organisations to benchmark their digital capability	Focus on technology with limited incorporation of organisational and human factors^21^

Information technologies (eg, computers) in health care have been implemented for decades, but advances in health care technologies and the data they produce (eg, mobile health, virtual care, precision medicine) are transforming care. Recouping the costs from an information technology investment in the short to medium term is unlikely due to the large upfront expense and the limited efficiencies that can be delivered in the short term. The traditional business case approach, which many existing digital health evaluation models adopt, fails to consider the value of the downstream effects of a connected digital health ecosystem, which enables sophisticated technological advances such as artificial intelligence, machine learning and precision medicine.

We suggest that digital health is no longer a technical capability for efficiency, but rather a critical enabler to achieving the quadruple aim. With the burden on the health care system to continue service delivery with static resourcing, an ageing population and growing health inequities, digital health provides an unrivalled opportunity to improve care at scale. The quadruple aim balances the economic costs (input side) with clinician experience (throughput) and health care quality and consumer experience (outputs).^6^ This is critical in the area of digital health, where a solitary focus on the deployment of health care technology has resulted in negative experiences and, in some cases, harm.^22^ Investing in digital health needs careful consideration, as technological advancements may cause unanticipated consequences, such as creating a digital divide,^23^ the depersonalisation of the clinician–patient relationship,^24^ poor integration with other health care systems,^1^, ^24^ and increased clinician workload.^1^ Modernising the frame of reference we use for assessing digital health impacts is critical and needs to be aligned to modern health care delivery underpinned by the quadruple aim.

## Case study: use of the quadruple aim for evaluating EMR implementations

Digitally mature health services are exploiting the capabilities of EMRs as a quality improvement platform, rather than solely information technology. Investments in hospital‐based EMRs are controversial, as they disrupt usual ways of working, can create slower workflows, and have been shown to contribute to clinician frustration and burnout.^24^, ^25^ Evidence supports the use of EMR capabilities such as clinical decision support systems, electronic medication management and digital clinical care pathways to improve care.^15^, ^26^ These capabilities are driven by the secondary use of routinely collected clinical data to improve the quality and safety of health care delivery, which has become an important enabler for health care improvement.^27^

The impact of EMR implementations over time can be described using a three‐horizon conceptual model of digital transformation (Box 2):

- horizon 1 focuses on building digital workflows to streamline the collection of clinical data;
- horizon 2 creates aggregated data and analytics for quality improvement; and
- horizon 3 implements new digitally enabled models of care.

Box 2 **Identified impacts of electronic medical records over a 10‐year time frame**

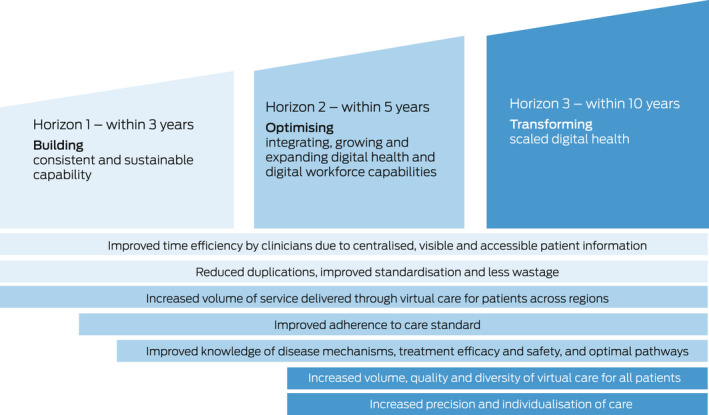

Traditional business cases evaluating the impact of hospital‐based EMRs only evaluate horizon 1 (the implementation of the digital workflows), neglecting the value of the aggregated data, analytics and the new models of care that develop over time.^28^

The immediate implementation impact is limited to easily measurable efficiencies (eg, reduction in printing costs).^28^ The increase in quality and safety enabled by data, analytics and new models of care in a digitally transformed health care system are realised in horizons 2 and 3, which are not captured in traditional business cases. We are therefore not adequately measuring the real long term impact of EMRs on health, patients, clinicians, health services, and our community.

In our case study, we also sought to understand the economic value of a hospital‐based EMR implementation. Economic value refers to the value an individual places (from both subjective and objective elements) on a good or service based on the benefit (perceived or actual) that they derive from it. It can be difficult to accurately measure the economic value of health care, where subjective constructs such as quality of life and patient experience are important elements.

## A new framework: a balanced view of digital health value

We previously undertook a comprehensive literature review to extract published metrics and evaluation methods for EMR implementations,^28^ and mapped these to the quadruple aim of health care. We then improved the evaluation framework, adapting existing economic approaches in other sectors and implementing a process of engagements including consultations with stakeholders across academia, government and industry.

The new framework we developed incorporates additional elements of value that do not yet have a market price but have a definite economic value, whether positive or negative, for the stakeholders (Box 3). This includes items such as workforce satisfaction, which can be negative in the short term due to disruption and positive in the long term due to learned improved work practices. The framework captures elements that are hypothesised to create benefits but require testing for confirmation (eg, savings from reduced primary care use after admission to hospital). Additional elements will emerge over time through system improvements, new technologies and evolving models of care.

Box 3 **Mapping the quadruple aim of health care to impacts of the electronic medical record (EMR)**

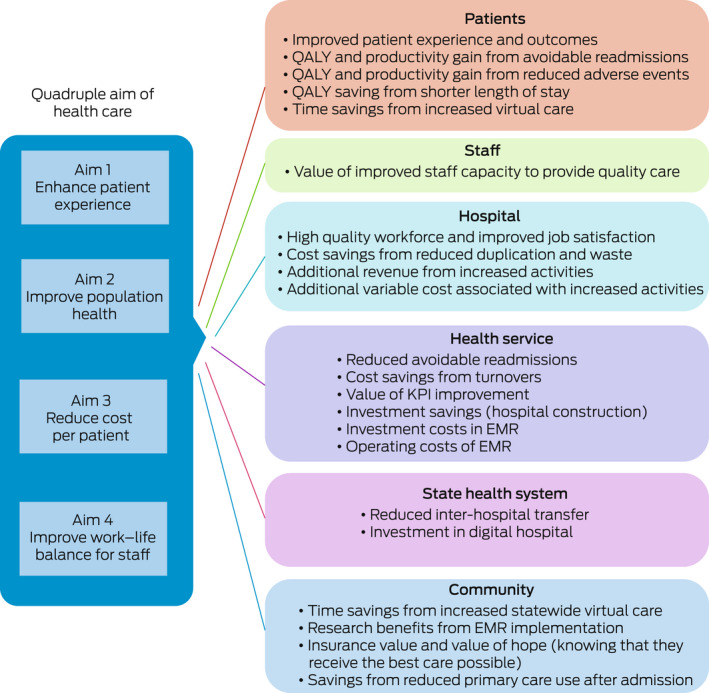

KPI = key performance indicators; QALY = quality‐adjusted life‐years.

## Conclusion

Health care organisations are currently not measuring the true value of digital health implementations and, thus, digital investment may not be prioritised given the competing health care demands. A modern, streamlined framework is critical to providing a balanced and long term view of the impact of digital transformation as it shifts away from an efficiency tool to a quality and safety improvement platform that enables innovation.

The impact of digital health interventions that are meaningful to staff, clinicians and consumers that goes beyond simplistic profit and loss evaluations needs to be acknowledged and measured. Focusing on financial outcomes alone is unlikely to deliver a comprehensive view of the value of digital health and will slow the necessary transformation of our increasingly unsustainable health care system. Clinicians should be aware that there is a balanced way to assess both positive and negative impacts of digital health implementations. This ensures that, in addition to productivity, non‐financial benefits such as quality of care, clinician experience and health outcomes are at the centre of the inevitable digital transformation of health care.

## Open access

Open access publishing facilitated by The University of Queensland, as part of the Wiley ‐ The University of Queensland agreement via the Council of Australian University Librarians.

## Competing interests

No relevant disclosures.

## Provenance

Not commissioned; externally peer reviewed.
